# Adaptive evolution of *SCML1 *in primates, a gene involved in male reproduction

**DOI:** 10.1186/1471-2148-8-192

**Published:** 2008-07-05

**Authors:** Hai-hui Wu, Bing Su

**Affiliations:** 1 State Key Laboratory of Genetic Resources and Evolution, Kunming Institute of Zoology, Chinese Academy of Sciences, Kunming, Yunnan, PR China; 2 Kunming Primate Research Center, Chinese Academy of Sciences, Kunming, Yunnan, PR China; 3 Graduate School of Chinese Academy of Sciences, Beijing, PR China

## Abstract

**Background:**

Genes involved in male reproduction are often the targets of natural and/or sexual selection. *SCML1 *is a recently identified X-linked gene with preferential expression in testis. To test whether *SCML1 *is the target of selection in primates, we sequenced and compared the coding region of *SCML1 *in major primate lineages, and we observed the signature of positive selection in primates.

**Results:**

We analyzed the molecular evolutionary pattern of *SCML1 *in diverse primate species, and we observed a strong signature of adaptive evolution which is caused by Darwinian positive selection. When compared with the paralogous genes (*SCML2 and SCMH1*) of the same family, *SCML1 *evolved rapidly in primates, which is consistent with the proposed adaptive evolution, suggesting functional modification after gene duplication. Gene expression analysis in rhesus macaques shows that during male sexual maturation, there is a significant expression change in testis, implying that *SCML1 *likely plays a role in testis development and spermatogenesis. The immunohistochemical data indicates that *SCML1 *is preferentially expressed in germ stem cells of testis, therefore likely involved in spermatogenesis.

**Conclusion:**

The adaptive evolution of *SCML1 *in primates provides a new case in understanding the evolutionary process of genes involved in primate male reproduction.

## Background

Proteins involved in sexual reproduction often evolve rapidly due to positive selection [1-4]. Although the selective forces are unclear, a variety of hypotheses have been proposed including mate choice, intra-sexual competition and sexual conflict, which are different forms of sexual selection. The rapid evolution of these proteins may contribute to several important biological aspects such as reproduction and speciation. It has long been recognized that gene duplication is a major source of genomic novelties. Therefore, the newly duplicated genes involved in reproduction are likely the targets of natural and/or sexual selection.

Using exon trapping, van de Vosse et al [5] identified a novel gene in human located on Xp22, named as SCM-like-1 (*SCML1*), which is similar with the Scm gene in Drosophila. In the human genome, *SCML1 *spans 18 kb and contains 8 exons. Northern blot analysis detected a major *SCML1 *transcript of approximately 3-kb in all human adult and fetal tissues tested [5].

*SCML1 *gene is a polycomb group (PcG) gene. Most of the PcG genes are expressed throughout embryonic, larval and pupal development, and are required continuously to maintain restricted homeotic expression in Drosophila. [6-10]. Most mammalian PcG genes have Drosophila homologs [11,12]. Compared to Drosophila, the mammalian PcG genes have acquired novel functions during evolution because PcG knockout mice exhibit numerous phenotypes including hematopoietic defects, neural crest defects, cardiac anomalies, and sex reversal [12,13]. *SCML1 *is likely a recently duplicated gene during mammalian evolution due to the absence of orthologs in Drosophila, zebrafish and chicken.

In the SCM family, there are other two genes, *SCML2 *and *SCMH1*, which have orthologs in all vertebrate species and are located on chromosome Xp22 [14] and chromosome 1p34 [15] respectively. *SCMH1 *is a core component of polycomb repressive complex 1 (PRC1) [16-18] which is involved in the maintenance of repression and can block chromatin remodeling[17], and it plays an important role in regulation of homeotic genes in embryogenesis[19]. *SCML2 *is also involved in PRC1's regulation[20]. A recent study showed that *SCML2 *is over-expressed in acute myeloid leukaemia, suggesting its role in differentiation and cell cycle regulation[21]. As *SCML2 *and *SCMH1 *are the ancient copies in the *SCM *family, they would serve as the ideal reference genes when dissecting the molecular evolution of *SCML1 *in primates.

Through a genome-wide comparison, we have identified 34 candidate genes including *SCML1 *that showed rapid nonsynonymous sequence divergence between human and chimpanzee [22], therefore an implication of adaptive evolution of these genes during primate evolution. To test whether *SCML1 *is the target of selection in primates, we sequenced and compared the coding region of *SCML1 *in major primate lineages, and we observed the signature of positive selection.

## Methods

### DNA samples

The major lineages of primates were sampled, including three great ape species (chimpanzee-*Pan troglodytes*, gorilla-*Gorilla gorilla *and orangutan-*Pongo pygmaeus*), two lesser ape species (white-browed gibbon-*Bunopithecus hoolock *and white-cheeked gibbon-*Nomascus leucogenys*), two Old World monkey species (rhesus macaque-*Macaca mulatta *and Yunnan snub-nosed monkey-*Rhinopithecus bieti*) and one New World monkey species (common marmoset-*Callithrix jacchus*). The common ancestor of the tested primate species can be traced back to about 45 million years ago [23]. All the DNA samples were from collection in Kunming Cell Bank of CAS and Kunming Blood Center in China.

### PCR and sequencing

All the samples were sequenced for the full length coding region of *SCML1*. Primers for all the primates were designed by aligning the published sequences of human (Esembl ID: ENSG00000047634) and macaque (Ensembl ID: ENSMMUG00000012899, Ensemble genome browser [24]). The primer sequences are listed [see Additional file 1].

PCRs were performed with rTaq under conditions recommended by the manufacturer (TAKARA Company). Sequencing was performed in both directions with forward and reverse primers using the BigDye terminator sequencing kit on an ABI 3130 automated sequencer. There are 8 exons in *SCML1 *gene, and the first exon is non-translational, therefore, not sequenced in this study. Overlapping chromatogram files retrieved from the sequencer were analyzed and edited using the SeqMan program in the Lasergene software package (DNASTAR Inc).

### Sequence analysis

The DNA sequences were aligned with the CLUSTALW program implanted in Mega [25,26] and checked manually. There are several in-dels (do not change the reading frame) in the coding region of common marmoset, and those sites were removed in the sequence analysis. The known phylogeny of primate species was used[23,27]. The ancestral sequences were inferred by PAML 3.15 [28]. The synonymous (*ds*) and nonsynonymous (*d*_*N*_) substitution rates of each branch were calculated with the use of the maximum likelihood method under the free-ratio model [28].

### Test of selection

Positive selection can be inferred from a higher proportion of nonsynonymous than synonymous substitutions per site (*d*_*N*_/*d*_*S *_> 1). To detect specific amino acid sites under positive selection, we applied the site models in the codeml program of the PAML package. Using this set of models, we obtained the log likelihood estimates (L) of a tree topology under models that impose alternative assumptions in terms of rate variation (ω = *dN*/*dS*) over different codon sites [29,30]. The model M0 was used to evaluate the general sequence substitution pattern of *SCML1 *in primates assuming a constant ω ratio across codon sites. M0 estimates the overall *ω *for the data. The M1a model estimates single parameter, *p*0, with *ω*0 = 0, and the remaining sites with frequency *p*1 (*p*1 = 1-*p*0) assuming *ω*1 = 1. We first compared model M0 with M1a to determine which model is more realistic for the data and M1a tuned out to be the better one. Then we compared model M2a (selection) and M1a (nearly neutral) to test if invoking of positive selection in model M2a would better explain the data [31,32]. It was suggested that under certain scenarios, a beta distribution of ω is more realistic, therefore, we also conducted the selection test by comparing model M7 and M8, in which a beta distribution of ωwas assumed. We also conducted a more stringent test by comparing M8 with M8a. The LRTs between nested models were conducted by comparing twice the difference of the log-likelihood values (2ΔL) between two models [32]. If the log likelihood test suggests the presence of sites under positive selection, we then identified these sites by using a Bayesian method to estimate posterior probabilities (P) [33].

### Comparative evolutionary analysis among SCML1, SCML2 and SCMH1

Sequences of *SCML2 *and *SCMH1 *genes were obtained using BLAST (GenBank and Ensembl) for five primate species including human, chimpanzee, orangutan, rhesus macaque and common marmoset. The sequence IDs are: *SCML2*, *Homo sapiens *(ENST00000398048), *Macaca mulatta *(ENSMMUG00000005084), *Pan troglodytess *(ENSPTRG00000021710); *SCMH1, Homo sapiens *(ENST00000326197), *Pan troglodytess *(ENSPTRG00000000601), *Macaca mulatta *(ENSMMUG00000017104). With the use of human *SCML2 *and *SCMH1*, we searched the genomes of orangutan and common marmoset with Blastn and obtained the coding sequences of these two genes[34].

Protein sequences were aligned with the CLUSTALW program implanted in Mega4 [26] and the ω calculation was conducted using the codeml program of PAML3.15 [32]. The ratios of *d*_*N *_and *d*_*S *_were estimated by using PAML3.15, and the Z test(data not show) was used to evaluate the ratio difference between each branches [26]. Similar neutrality tests described above were used in comparing the evolutionary patterns among the three genes.

RT-PCR analysis RNAs were extracted using the Tri-Reagent kit based on the manufacturer's specifications (Invitrogen Inc.). For gene expression analysis of rhesus macaques during development, a total of 20 testis samples were analyzed including ten 1–2 year old monkeys (sexually immature) and ten 7–8 years old monkeys (sexually matured). The T test was used for statistical evaluation of expression difference.

For tissue expression analysis in rhesus macaques, a total of 12 tissue types (1–2 year old male macaques) were analyzed including brain, cartilage, heart, large intestine, small intestine, liver, lung, muscle, pancreas, spleen, stomach and testis. All the rhesus macaque tissue samples were collected from the Kunming Primate Research Center, Chinese Academy of Sciences.

For real-time quantitative RT-PCR analysis, cDNAs were synthesized with SuperScript™ III (Invitrogen) from 5 μg of total RNA in a total volume of 20 μl with oligo(dT) primer in accordance with the manufacturer's instructions. SYRB Green I-based real-time PCR was carried out using the DNA Engine Opticon^® ^2 Continuous Fluorescence Detection System (MJ, BioRad). After an initial denature step for 5 min at 94°C, conditions for cycling are 40 cycles of 20 sec at 94°C, 20 sec at 58°C, 20 sec at 72°C. At the end of the PCR cycles, a melting curve was generated to identify specificity of the PCR product. For each run, serial dilutions of rhesus macaque GAPDH (glyceraldehyde-3-phosphate dehydrogenase) plasmids were used as standards for quantitative measurement of the amount of amplified DNA. In addition, for normalization of each sample, mGAPDH primers were used to measure the amount of GAPDH cDNA. All samples were run in triplicates and the data were presented as a ratio of *SCML1*/GAPDH. The ΔCt values were calculated and then converted into the linear-scale expression levels. Oligonucleotides were obtained from Invitrogen. Negative controls were performed with water as template. The primer sequences are:

GAPDH F primer 5'ACTTCAACAGCGACACCCACTC3'

GAPDH R primer 5'CCCTGTTGCTGTAGCCAAATTC3'

*SCML1 *F primer 5'CTCCTACCCTGAAAGTTATAGCC3'

*SCML1 *R primer 5'TCTGAGGGATGCACTGGAC3'

### Immunohistochemical analysis

The liquid nitrogen stored tissue was sectioned (10 μm) using a HM550 tissue processor (Microm). The frozen section slides were stored at -80°C in a sealed slide box. Sections were stained using the standard immunohistochemical method. The mouse monoclonal antibodies generated using human *SCML1 *protein (dilution 1:100, Abnova) and the goat anti-mouse IgG antibody (dilution 1:200, Bethyl) were used following the manufacturer's instruction. The negative control used is the buffer-only samples with no mouse antibodies. Immuno-reactivity was visualized by using 0.025% 3.3'-diaminobenzidine tetrachloride/0.001% H_2_O_2_. These slides were washed with phosphate-buffered saline (pH 7.4). The sections were counterstained with hematoxylin for a few seconds.

## Results

### Sequence substitution pattern of SCML1 in primates

**Figure 1:**
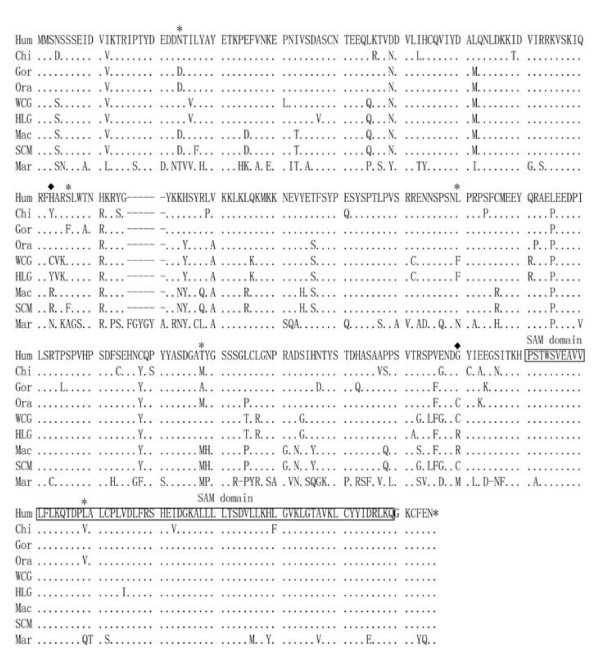
**Protein sequence alignment of *SCML1 *in human and nonhuman primates.** The SAM domains are indicated. The SAM domain is 64 amino acids in length. Sterile alpha motif (SAM) domains are known to exhibit diverse protein-protein interaction modes[57] (Hum: human – *Homo sapiens*, Chi: *Pan troglodytes*, Gor: *Gorilla gorilla*, Ora: *Pongo pygmaeus*, HLG: white-browed gibbon – *Bunopithecus hoolock*, WCG: white-cheeked gibbon – *Nomascus leucogenys*, Mac: rhesus monkeys – *Macaca mulatta*, and SCM: Yunnan snub-nosed monkey – *Rhinopithecus bieti*, Mar: common marmoset – *Callithrix jacchus*.) The sites under positive selection are highlighted including 23N, 95S, 153L, 201T, 270L (P > 95%, labeled with *) and 92H, 242G (P > 99%, labeled with ◆). The sequence IDs are Hum: EU370780, Chi: EU370781, Gor: EU370782, Ora: EU370783, WCG: EU370784, HLG: EU370785, Mac: EU370786 and SCM: EU370787. The marmoset's ortholog of *SCML1 *was obtained through blast search [34].

A total of nine primate species were sequenced covering the complete 990–1,002 bp coding region of *SCML1*. There are 215 sites (21.98%, 215/978, in-dels were not counted) showing sequence substitutions in the nine primate species tested. When translated into protein sequences (329–333 amino acids, Figure 1), there are 129 sites (38.51%) having substitutions, an implication of rapid sequence changes during primate evolution. For example, the protein sequence substitution rates (measured by *d*_*N*_) of *SCML1 *between human and Old World monkeys are relatively fast (human vs. rhesus monkey, 0.045; human vs. Yunnan snub-nosed monkey, 0.055) among those male reproduction associated genes in primates [35].

### Test of selection on SCML1 in primates

**Figure 2:**
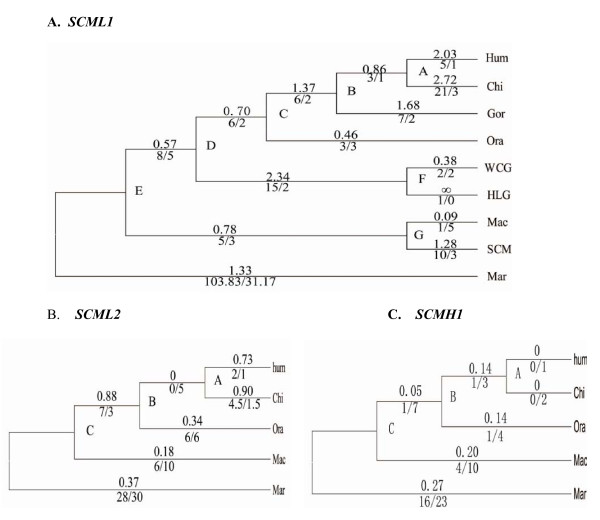
**Molecular evolution of *SCML1, SCML2 *and *SCMH *reconstructed over the known phylogenetic tree of primates.** A) The ω values of different lineages in primates were calculated using the *SCML1 *coding sequences. The phylogeny was drawn roughly to the scale of evolutionary time. ∞ refers to ω value with *dS *being zero. B, C) The phylogenetic trees of *SCML2 *and *SCMH1*. The ω values are shown above each branch. The numbers of nonsynonymous vs. synonymous substitutions (N/S) are shown below each branch.

We calculated the *d*_*N*_/*d*_*S *_ratio (also called ω), which measures the rate of protein evolution as scaled to mutation rate for all the branches. We also obtained the numbers of nonsynonymous and synonymous substitutions for each branch (Figure 2A) [36]. As shown in Figure 2A, most of the primate lineages have large ω values (ω > 1) except for orangutan and rhesus macaque, again suggesting rapid amino acid changes during primate evolution.

The rapid protein sequence evolution can be explained either by Darwinian positive selection or by relaxation of negative (or purifying) selection. To test the hypothesis that they could be under Darwinian positive selection, we conducted the analysis for positive selection at individual amino acid sites using maximum likelihood models by estimating ω values[28,29,37]. The results are presented in Table 1. We first conducted the analysis using M0. In the M0 analysis, the log likelihood L is -2619.84, and the estimated ω = 1.169, implying that there are varied evolutionary forces acting on the amino acid sites of *SCML1 *(neutral, negative selection and/or positive selection). Model M1a (nearly neutral) assumes two site classes in the sequence (0 < ω < 1 and ω1 = 1 fixed), and is significantly better than M0 (2ΔL = 13.26, P = 0.0002). Therefore, we use M1a as the null hypothesis in detecting selection.

**Table 1:** Neutrality tests of *SCML1 *in primates using maximum likelihood estimates (site-model)

Model	lnL	Estimates of parameters	2ΔlnL	Positively selected sites
M0:	-2619.84	ω = 1.169		None
M1a	-2613.21	p0 = 0.278 p1 = 0.722	13.26(1)**	Not allowed
M2a	-2599.55	p0 = 0.217 p1 = 0.660 p2 = 0.123 ω2 = 5.25	26.52(2)**	23N 153L 201T (95 =< P < 99%) 92H 242G (P > 99%)
M7	-2616.5	p = 1.096 q = 0.005 p0 = 0.892 p = 0.020		Not allowed
M8	-2599.65	q = 0.005(p1 = 0.108) ω = 5.78	33.9(3)**	3N 95S 153L 201T 270L (P > 95%) 92H 242G (P > 99%)
M8a	-2613.21	p0 = 0.278 p = 0.005 q = 1.728 (p1 = 0.722) ω = 1.0	27.3(4)**	Not allowed

Note: * refers to P < 0.05. ** refers to P < 0.01; (1): M1a versus M0, (2): M2a versus M1a, (3): M8 versus M7 (4): M8 versus M8a. The proportions of sites under positive selection and selective constraint are *p*1 and *p*0 respectively. The parameters *p *and *q *are used for the beta distribution *B*(*p*, *q*).

We next compared M1a and M2a (selection model), and M2a fits the data significantly better than M1a (2ΔL = 26.52 P < 0.0001), a strong signature of positive selection on *SCML1 *in primates. M2a suggests that 12.3% of the sites are under positive selection with ω_2 _= 5.25. In addition, to avoid the potential bias caused by the assumed substitution pattern in M1a and M2a, we also conducted the selection test by comparing M8 (selection model) and M7 (neutral model), in which a beta distribution for ω over sites was assumed. M8 provides significantly better fit to the data than M7(2ΔL = 33.9, P < 0.0001), again suggesting positive selection on *SCML1 *in primates. M8 suggests that 10.8% of the sites were under positive selection with ω = 5.78. Interestingly, M8 demonstrates a U-shaped distribution of beta values, suggesting that most sites are either highly conserved with dN/dS close to 0 or nearly neutral with dN/dS = 1, and only a small percentage of the sites were under positive selection. A more stringent test comparing M8 and M8a also supports the proposed positive selection (2ΔL = 27.3, P < 0.0001). The positively selected sites are shown in Table 1 and Figure 1[33,38]. Collectively, all the tests on selection can be better explained by the evolutionary model that invokes positive selection in primates.

### Evolutionary pattern comparison between SCML1, SCML2 and SCMH1

**Figure 3:**
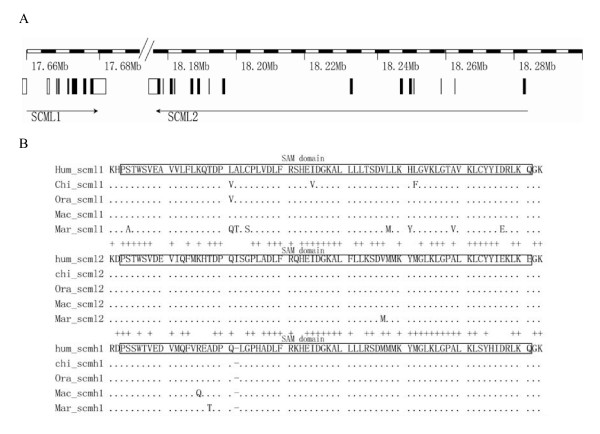
**Gene structure and sequence comparison among *SCML1*, *SCML2 *and *SCMH1*.** A) *SCML1 *and *SCML2 *are both located on the X-chromosome and the transcription directions are indicated by the arrows. B) The protein sequence alignment among the three genes. The SAM domains are highlighted. Sterile alpha motif (SAM) domains are known to exhibit diverse protein-protein interaction modes[57]

As *SCML1 *is likely a recent duplication during mammalian evolution, we compared the evolutionary patterns between *SCML1 *and the two other members of the SCM family, *i.e. SCML2 *and *SCMH1*, which are located on chromosome X and chromosome 1 respectively. The sequence alignment is showed in Additional file 2. *SCML1 *and *SCML2 *are only 15-kb apart on chromosome X (Figure 3A), and the intron and exon structure at the C terminus are highly conserved between them but with totally different N terminus. The SAM domain (sterile alpha motif) is highly conserved among all the three genes (Figure 3B). Strikingly, when comparing the amino acid sequences of the SAM domain among different primate species, *SCML1 *have much more between-specie amino acid changes than those of *SCML2 *and *SCMH1*. In the M0 analysis, the ω values for *SCMH1 *and *SCML2 *are 0.179 and 0.325 respectively, which are much smaller than that of *SCML1 *(ω = 1.169). This is an average over all sites in the protein and all lineages in the tree, therefore, suggesting a dominant role of purifying selection in the evolution of *SCML2 *and *SCMH1*. With the use of free ratio model, we calculated the *d*_*N*_/*d*_*S *_values of each primate lineages for *SCML2 *and *SCMH1*, and all the lineages show strong functional constraint (purifying selection) or neutral evolution (*d*_*N*_/*d*_*s *_≤ 1) (Figure 2B, 2C). The expression pattern of the three genes in human is different from each other though all of them are highly expressed in testis [see Additional file 3] implying functional divergence after gene duplication.

### Expression analysis of SCML1

**Figure 4:**
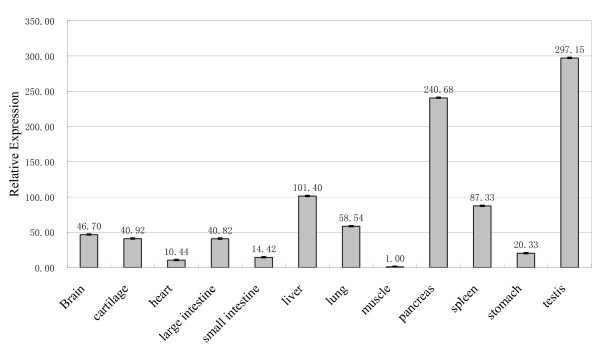
**The relative expression levels of *SCML1 *in different tissues.** A 1–2 year old male rhesus monkey was sampled and tested using real time quantitative PCR. The pancreas and testis showed the highest expression of *SCML1*. The numbers above the columns are the relative levels of expression.

Having shown that the evolution of *SCML1 *in primates is consistent with positive selection, by dissecting its expression pattern, we attempt to understand the driving force and the functional consequence of selection. We first detect the expression pattern of *SCML1 *by testing 12 different tissue types in 1–2 year old macaques and the result is shown in Figure 4. Testis and pancreas have the highest expression when compared with the other tissues. In human, according to the micro-array expression data, *SCML1 *is also preferentially expressed in testis [39]. The abundant expression of *SCML1 *in testis confirms its involvement in male reproduction. However, in humans [see Additional file 3], liver, fetal liver, and pituitary all show higher expression than pancreas, which is different from the expression pattern of rhesus macaque, implicating functional modifications of *SCML1 *during primate evolution. This is consistent with the proposed positive selection on *SCML1 *in primates.

**Figure 5:**
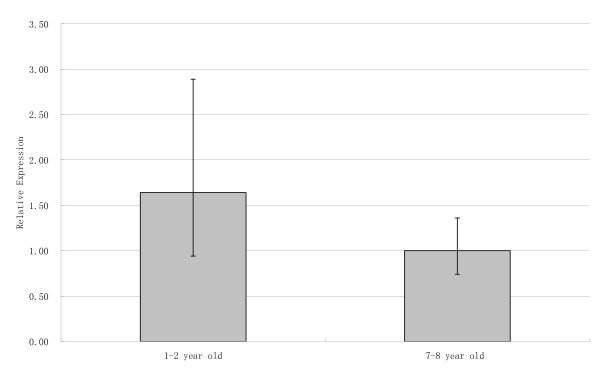
**The relative expression levels of *SCML1 *in testis during male sexual maturation, which was tested using real time quantitative PCR.** Two age groups were tested including the 1–2 year old macaque group and the 7–8 year old macaque group. Ten individuals were sampled for each group.

**Figure 6:**
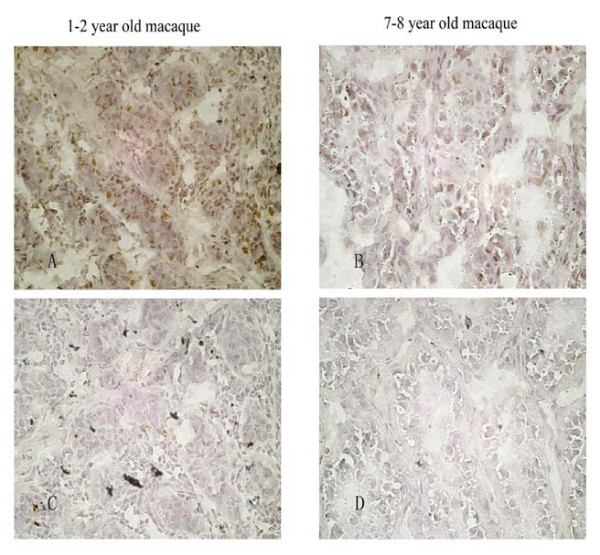
**The *in-situ *immunohistochemical analysis of *SCML1 *during testis development.** Both the 1–2 year old (A, C) and 7–8 year old macaques (B, D) were tested (40×). The expression of *SCML1 *is mostly located in spermatogonial stem cells (1–2 year old), and spermatogonia (7–8 year old). C and D are the controls without adding *SCML1 *mouse antibodies.

We then detect the potential expression change of *SCML1 *during male sexual maturation and a significant change was detected in testis. We compared two age groups, *i.e. *1–2 year old monkeys (sexually immature) and 7–8 year old monkeys (sexually matured). The result indicates that both age groups have abundant expression of *SCML1*, and there is a significant reduction (about 60%) in the adult group (p = 0.015, two-tailed t test) (Figure 5). We next carried out immunohistochemical analysis of the two age groups (Figure 6). The result shows that *SCML1 *is preferentially expressed in the germ stem cells of testis (spermatogonial stem cells in 1–2 year old monkeys, and spermatogonia in 7–8 year old monkeys) [40-48], and again the 1–2 year old group has higher expression than the adult group. The higher expression in the 1–2 year old monkeys is probably due to the relative abundance of stem cells at the sexually immature stage. The preferential expression in the stem cells of testis suggests that *SCML1 *is likely involved in spermatogenesis during sexual maturation in rhesus macaque.

## Discussion

We demonstrate that *SCML1 *evolves rapidly in primates, which was caused by Darwinian positive selection. Genes expressed exclusively or preferentially in testis are likely involved in male reproduction and have been shown to evolve rapidly under positive selection in previous studies ([1-3,49-55]. Our observation of rapid evolution in *SCML1 *provides another example of male reproductive gene under Darwinian positive selection in primates.

Darwinian positive selection may lead to functional changes of the target genes during evolution. The SAM domain located in the C terminal (Figure 1) is the only known functional domain of *SCML1*[56]. Among primates, the amino acid sequences of the SAM domains are relatively conserved across species and there is one site under positive selection (Figure 1). The SAM domain is known to exhibit diverse protein-protein interaction modes, and is involved in developmental regulation [57]. Through functional domain prediction [56-63], besides of the SAM domain, we identified a total of six fragments[64,65] (amino acid position 1–9, 13–23, 72–79, 88–114, 125–157 and 224–239) in *SCML1 *containing potential functional domains. For example, the fragment 1–9 is a signal peptide. The positively selected sites using 95% cutoff are listed in Table 1, and most of them are also located in the potential functional domains other than the structural domains (4/1 and 5/2 for model M2a and model M8 respectively). This distribution bias of the positively selected sites indicates that Darwinian positive selection on *SCML1 *targets the putative functional domains, which is consistent with the proposed functional modification of *SCML1 *during primate evolution.

Sexual selection is the favored explanation for the observed adaptive evolution of male reproductive genes [35,66]. The immunohistochemical and RT-PCR data suggests that *SCML1 *is important for the development and normal function of testis in primates. Therefore, it is reasonable to propose that the adaptive evolution of *SCML1 *in primates is likely due to sexual selection[4]. Sperm competition, one of the major mechanisms for sexual selection has been used to define the driving force of selection in promiscuous species, *e.g. *chimpanzee and human, which seems to explain the observed adaptive evolution of *SCML1 *since both chimpanzee and human are among the rapidly evolving lineages (ω > 1, Figure 2A). However, gibbon is a monogamous species with a high ω value, and the highly promiscuous rhesus monkey does not show accelerated evolution (ω = 0.09). Therefore, the branch-specific rapid evolution of *SCML1 *in primates does not provide consistent support for the sexual selection hypothesis. Other evolutionary mechanisms need to be tested, *e.g. *speciation [67-70].

The origin of *SCML1 *probably occurred at the early stage of mammalian radiation about 100 million years ago because we do not identify *SCML1 *in non-mammalian species, neither in mouse and rat, but in dog, cow and primates. The comparison of evolutionary and expression patterns among the three genes of the same *SCML *family suggests that the rapid evolution of *SCML1 *likely led to function modification of testis development and spermatogenesis in primates [71-73].

## Conclusion

The adaptive evolution of *SCML1 *in primates provides a new case in understanding the evolutionary process of genes involved in primate male reproduction.

## Abbreviations

Hum: human – *Homo sapiens*; Chi: chimpanzee – *Pan troglodytes*; Gor: gorilla – *Gorilla gorilla*; Ora: orangutan – *Pongo pygmaeus*; HLG: white-browed gibbon – *Bunopithecus hoolock*; WCG: white-cheeked gibbon – *Nomascus leucogenys*; Mac: rhesus monkeys – *Macaca mulatta*; SCM: Yunnan snub-nosed monkey – *Rhinopithecus bieti*; Mar: common marmoset – *Callithrix jacchus*.

## Authors' contributions

BS and HW conceived the project. HW executed the sequencing, data analysis, immunohistochemical analysis, real-time quantitative RT-PCR, data mining and statistical analysis. BS supervised the project execution. BS and HW wrote the manuscript.

## Supplementary Material

Additional file 1 The PCR primer sequences for *SCML1*. Click here for file

Additional file 2 The protein sequence alignment of *SCML2 *and *SCMH1*. Click here for file

Additional file 3 The expression patterns of *SCML1*, *SCML2 *and *SCMH1 *in normal human tissues. Click here for file
