# Supplementary material for: Adaptive evolution of SCML1 in primates, a gene involved in male reproduction
Source: BMC Evol Biol. 2008 Jul 5;8:192. doi: 10.1186/1471-2148-8-192 (PMC2459175; doi:10.1186/1471-2148-8-192)
Supplement: Additional file 1 — The PCR primer sequences for SCML1. [file 1471-2148-8-192-S1.doc]

The PCR primer sequences for *SCML1*.

| Primers | Sequence 5’🡪 3’ |
| --- | --- |
| *SCML1* E2F1 | AACCCAAAGTCCAATTCTATTTG |
| *SCML1* E2R1 | TATGGCAGATTCTCACAGAGTT |
| *SCML1* E2F2 | AATTCAACCCAAAGTCCAAT |
| *SCML1* E2R2 | CACTTTGAAAAACTGTATGGCA |
| *SCML1* E3F1 | TAAGTGTAGTTGGGTTAATGAG |
| *SCML1* E3R2 | GGGAAAGTAAATTGGTTAAGTG |
| *SCML1* E3F2 | AGCATATAAAGGGTTAAGCTCAG |
| *SCML1* E3R2 | TCAAGGGGTTTCAGAAGTCTTA |
| *SCML1* E3R1 | TCAGAAGAATCCAGGTTTAGAGT |
| *SCML1* E4F1 | AAACCTGGATTCTTCTGATATAATA |
| *SCML1* E4R1 | ACAATGGGATGATAATCATAA |
| *SCML1* E4F2 | CCCCTTGAAAAGTTGGTG |
| *SCML1* E4R2 | CACAATGGGATGATAATCATAAA |
| *SCML1* E5F1 | GCCAGGCCTTGAATAGGA |
| *SCML1* E5R1 | CAAACCTAAACTTCAGCGTTCTC |
| *SCML1* E5F2 | AGCAGGGGAGGGGACTACT |
| *SCML1* E5R2 | ATCAAACCTAAACTTCAGCGTTC |
| *SCML1* E6F | AAACTCTGTTGGGAGAACGCTGAA |
| *SCML1* E6R1 | TGGGGTCATTATGAGTATAAGTGGAG |
| *SCML1* E6R2 | GCAGTGCCTTGCACATAATATGC |
| *SCML1* E7F | AGCATTCCTCCTTACCTAACATCTC |
| *SCML1* E7R | TGTATTTTAGGCACTTCTACACCCA |
| *SCML1* E8F | ACCAAACGGTGCTTCACTGTAC |
| *SCML1* E8R | CGAGATGAACCAGAATCAAAGG |
| *SCML1* E8R2 | AAACAAAAACGACTAAAGGGCAGA |
