# Supplementary material for: Adaptive evolution of SCML1 in primates, a gene involved in male reproduction
Source: BMC Evol Biol. 2008 Jul 5;8:192. doi: 10.1186/1471-2148-8-192 (PMC2459175; doi:10.1186/1471-2148-8-192)

The protein sequence alignment of *SCML2* and *SCMH1.* The alignment was done by Mega4 [26]. The sign of “+” indicates the same sequence between *SCML2* and *SCMH1*. And the sign of “–“ indicates the gap. (the start codon was removed).


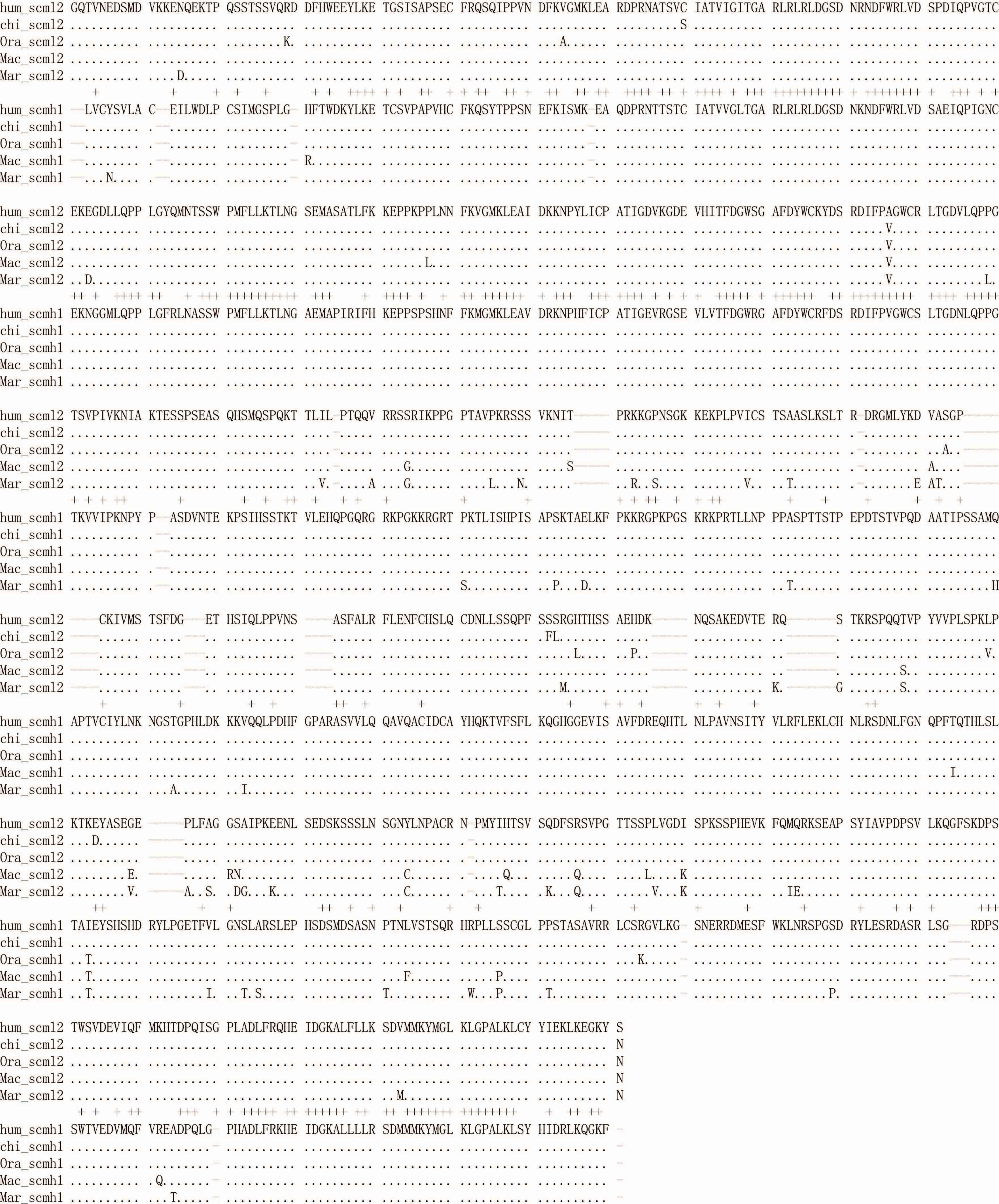

Supplement: Additional file 2 — The protein sequence alignment of SCML2 and SCMH1. [file 1471-2148-8-192-S2.doc]
