# Supplementary material for: Adaptive evolution of SCML1 in primates, a gene involved in male reproduction
Source: BMC Evol Biol. 2008 Jul 5;8:192. doi: 10.1186/1471-2148-8-192 (PMC2459175; doi:10.1186/1471-2148-8-192)
Supplement: Additional file 3 — The expression patterns of SCML1, SCML2 and SCMH1 in normal human tissues. [file 1471-2148-8-192-S3.doc]

The expression patterns of *SCML1*, *SCML2* and *SCMH1* in normal human tissues. The histogram shows the relative mRNA expression of *SCML1, SCML2* and *SCMH1* in the normal tissues of human, and the data was from published microarray analysis[74].
